# Order is needed to promote linear or quantum changes in nutrition and physical activity behaviors: a reaction to 'A chaotic view of behavior change' by Resnicow and Vaughan

**DOI:** 10.1186/1479-5868-3-29

**Published:** 2006-09-19

**Authors:** Johannes Brug

**Affiliations:** 1 ErasmusMC University Medical Centre Rotterdam, Department of Public Health, P.O. Box 2040; 3000 CA Rotterdam, the Netherlands

## Abstract

Recently, Drs. Ken Resnicow and Roger Vaughan published a thought-provoking paper in the International Journal of Behavioral Nutrition and Physical Activity (IJBNPA). They argue that the most often used social-cognition theories in behavioral nutrition and physical activity are of limited use. These models describe behavior change as a linear event, while Resnicow and Vaughan posit that behavior change is more likely to occur in quantum leaps that are impossible to predict. They introduce Chaos Theory into the behavioral nutrition and physical activity domain as a more valid framework to study the complex process of health behavior change. The present paper is a commentary on Resnicow and Vaughan's article by Resnicow's opponent in a recent debate-session at the annual meeting of the International Society of Behavioral Nutrition and Physical Activity. The chair of that meeting, Prof. Tom Baranowski, provides a separate commentary on Resnicow and Vaughan's paper also published recently in the IJBNPA. In the present commentary I relate Resnicow and Vaughan's paper to the other contributions to the Theory debate in the IJBNPA. I recognize the limited success of social cognition models, and, next to a better application of these models and more thorough research to test these model, also support research to further test the quantum and chaotic character of health behavior change. However, if such research supports the chaotic and quantum nature of health behavior change, the implications for behavioral nutrition and physical activity interventions may be limited, because even if behavior change is quantum rather than linear, the social cognition models are still relevant to inform interventions to promote quantum leaps in behavior change.

## Background

From the very start of the International Journal of Behavioral Nutrition and Physical Activity (IJBNPA) the journal has recognized the limited success of the prevailing health behavior theories in explaining and predicting nutrition and physical activity behaviors and behavior change. The IJBNPA therefore encouraged a theory debate to promote a discussion on the limitations of common behavioral theories, and ways to improve the theories and how the theories are used. This in order to better inform behavioral nutrition and physical activity interventions. Four theory debate papers had been published so far. Dr. Robert Jeffery did the kick-off with a straightforward critique of the social cognition theories that are most often used to explain and predict nutrition and physical activity behaviors, such as the Theory of Planned Behavior, Health Belief Model, protection Motivation Theory and Social Cognitive Theory [1]. Jeffery argued that these theories focus primarily on psychological variables as predictors of people's motivation to change, but often fail to address people's abilities and opportunities to change. He further described a number of projects to promote weight loss he was professionally involved in, to show that using these theories to inform behavioral nutrition and physical activity interventions did not improve the chances of success. Jeffery further argued that a focus on the interface between the person and the environment as well as a return to the more classic learning theories might better inform weight loss interventions.

In the second theory debate paper [2], Dr. Rothman also concluded that the prevailing theories have limited success in explaining behavior change. Although he recognizes the importance of structured protocols to better apply theoretical constructs in intervention development, he argued that such protocol may only help to provide better guidance as to how existing theories should be applied, while more effort is necessary to refine, expand or reject existing theories. These efforts should be based on intervention studies rather than observational studies [2], or, in the words of Robinson and Sirard, on solution-oriented instead of problem-oriented research [3].

In the third theory debate paper I collaborated with two colleagues to build upon these two excellent contributions [4]. We argued, in line with Jeffery, that the leading behavioral theories focus too much on motivation only, and we agreed with Rothman that more experimental research is necessary to test these theories in the behavioral nutrition and physical activity domain, and that the prevailing theories should not be viewed as fixed entities, but should be further refined as well as integrated. However, we also argued that lack of supporting evidence for the prevailing theories might be due to the improper way in which these theories are often used to inform interventions and not because of the invalidity of the theories. In the fourth theory debate paper, Kremers and colleagues proposed an integrative theoretical framework, specifically for energy-balance-related nutrition and physical activity behaviors. This framework explicitly includes the interface between person and environment that Jeffery missed in the social cognition models he criticized, and the framework addresses behavior change instead of motivation, paying attention to possible mediators and moderators in determination of behavior changes, and thus focusing on hypothetical causal pathways instead of associations [5].

Now Drs. Ken Resnicow and Roger Vaughan [6] provide a more radical contribution to the theory debate. While the former contributors appear to agree that the prevailing theories are of value, and should be refined or extended, Resnicow and Vaughan argue that the foundation of the prevailing theories is invalid, and that a fundamental paradigm shift in health behavior theory is needed.

## Discussion

Resnicow and Vaughan [6] present two key arguments why the prevailing behavior theories, such as the Theory of Planned Behavior, Social Cognitive Theory, and the Transtheoretical Model, are of limited use for healthful nutrition and physical activity promotion: (I) behavior change is influenced by such a complex set of interacting variables that it should be viewed as a chaotic system; (II) behavior change does not follow a linear pattern but rather occurs with 'quantum leaps'. I will briefly discuss both these issues, before addressing the consequences of the paradigm shift that Resnicow and Vaughan propose.

The complexity of nutrition and physical activity behavior change.

The fact that nutrition and physical activity behaviors are influenced by a complex set of interrelated variables is well recognized. First of all, as others and I argued in earlier contributions to the theory debate in the IJBNPA [4,5], nutrition and physical activity are not behaviors but rather behavioral categories, i.e. complex collections of specific behaviors [7]. Although the present day obesity epidemic suggests otherwise, people cannot eat an unlimited amount of food, and people have only a limited amount of time, so different specific nutrition behaviors and different sedentary and physical activity behaviors are not independent but may cluster, interact or compete, and each of these specific behaviors will have its own specific determinants.

I have argued, following a framework suggested by Rothshild [8], that these determinants can be categorized in three groups: motivation, abilities and opportunities [4,9]. Such a simplification in categories at least helps me to, for example, explain to students and health promotion practitioners that inducing behavior change is not merely a case of knowledge transfer, increasing risk perceptions, or otherwise convincing people that the pros of change outweigh the cons, but that teaching and practicing skills as well as environmental changes are often very much needed to get people to change their nutrition or physical activity behaviors. However, helpful as it may be, categorization of a complex set of determinants does not change the complexity and I very much agree with Resnicow that for many nutrition and physical activity behaviors, let alone public health-relevant behavioral categories, mapping a complete picture of mediation, moderation [10,7], and clustering [11,12] of all potential determinants is often impossible [5]. Recently Glass and McAtee [13] proposed a multilevel framework for the study of obesity-related health behaviors that also nicely illustrates the complexity of the issue. Resnicow posits that Chaos Theory truly recognizes such complexity and may therefore provide a better framework for understanding health behavior change.

Behavior change in quantum leaps instead of linear fashion.

Resnicow and Vaughan also argue that a key characteristic of the prevailing health behavior theories is invalid; the prevailing theories explicitly of implicitly treat behavior change as a linear event. According to Resnicow and Vaughan behavior change is much more likely to occur in 'quantum leaps'; 'mini-epiphanies' and 'tipping points' may occur that induce radical changes in behavior. Whether and when such dramatic changes occur are "virtually impossible to predict", and they may be heavily sensitive to initial conditions. Adopting this line of thinking makes nutrition and physical activity behavior change a random process.

I find Resnicow and Vaughan's contribution to the theory debate very useful. It forced me to rethink behavioral nutrition and physical activity theory and intervention approaches; based on the reactions to the theory debate key note at this year's ISBNPA conference, where Resnicow introduced his arguments, I was not the only one. Despite the strength of their arguments, I am not yet convinced. First of all, I want to see evidence. I agree that the scientific evidence for the prevailing health behavioral theories may be weak for many nutrition and physical activity behaviors. However, the direct empirical evidence for a chaotic nature of such behaviors or the quantum leap character of behavior change is absent. The authors do refer to evidence for quantum change in alcohol dependency behaviors, but such addictive behaviors may be different from nutrition and physical activity, i.e. more complex behavioral categories for which cessation is certainly not recommended. A next step to further build Resnicow and Vaughan's framework would therefore be to propose specific hypotheses to test the value of the framework. First of all, an empirical study to test whether nutrition and physical activity behavior change indeed occurs in quantum leaps, initiated by 'mini-epiphanies' and associated with 'tipping points' [6] rather than in a linear fashion, is certainly possible.

Furthermore, there are empirically supported arguments why we should not disregard existing behavioral theories in order to embrace chaos.

First, certainly not all nutrition and physical activity behaviors are so complex that a Chaos Theory approach should be considered. For example, children's nutrition behaviors in schools are strongly dependent on what they can bring to school and on what is provided within schools; changing the school environment will directly influence what children eat at schools. Bere et al., for example, showed that cost free provision of fruits and vegetables in schools led to increased fruit and vegetable intakes among school children [14]. Similarly, children's physical activity during school hours is strongly dependent on how many hours of physical education are provided, and increasing time for physical education does lead to increased physical activity levels [15,16].

Second, the practical implications of the Chaos Theory paradigm seem limited, and I believe that Resnicow and Vaughan would agree that behavioral nutrition and physical activity interventions still ask for insight in people's motivations, abilities and opportunities, despite a possible chaotic nature of these behaviors. Resnicow and Vaughan propose some practical implications based on the chaotic and quantum character of health behavior change. They use the analogy of "ping-pong balls in a lottery machine" to illustrate what they mean: the role of health communications may be similar to spinning the lottery machine where ping pong balls that represent different motivations, abilities and beliefs in opportunities; health communicators should try to spin that machine periodically under various circumstances to improve the chances that the spinning will result in a mass-epiphany or a tipping point. But how should we spin the lottery machine? Should the spinning, i.e. the medium, strategy and content of health communications, be random? To me it still makes sense to have evidence-based insight in which motivations, abilities and self-efficacy beliefs are on those ping-pong balls, in order to target or tailor the machine spinning to this 'initial condition', and to improve the chances that the intervention is regarded as personally relevant which improves the chances that the health communications get sufficient attention [17,18].

## Conclusion

The paper by Drs. Ken Resnicow and Roger Vaughan presents a thought provoking contribution to the 'theory debate'. I fully agree with their arguments that nutrition and physical activity behaviors are often influences by a complex set of variables, which makes prediction of behavior change very difficult if not impossible. I also agree with their implicit argument, and the explicit arguments made by other in the ongoing theory debate in the IJBNPA, that behavioral nutrition and physical activity theory should rise above application of social cognition models. Furthermore, their hypothesis that behavior change occurs in a quantum rather than a linear fashion, and the argument that Chaos Theory may be a better framework to study health behavior change than the 'usual suspect' linear health behavior theories should lead to further discussion as well as empirical research to test some of the propositions made in Resnicow and Vaughan's paper.

However, the practical implications for behavioral nutrition and physical activity interventions seem limited. I still firmly believe in a planned approach to behavioral nutrition and physical activity interventions [4], which implies that we should identify relevant and modifiable determinants or mediators of behavior change, including (but not limited to) social cognitions that form the backbone of the prevailing health behavior theories. In an earlier contribution to the theory debate [4], I already argued that we should not throw away these prevailing theories before we have given them a fair change by applying them to behavior change instead of to behavior states, by testing the theories or theoretical constructs in controlled intervention studies instead of observational studies, and by problem- and behavior-specific integration of insights from different theories [19].
